# Negative Regulation of EGFR/MAPK Pathway by Pumilio in *Drosophila melanogaster*

**DOI:** 10.1371/journal.pone.0034016

**Published:** 2012-04-13

**Authors:** Sung Yun Kim, Ji Young Kim, Sumira Malik, Wonseok Son, Ki-Sun Kwon, Changsoo Kim

**Affiliations:** 1 Hormone Research Center, School of Biological Sciences and Technology, Chonnam National University, Yongbong-Dong, Gwangju, South Korea; 2 Aging Research Center, Korea Research Institute of Bioscience and Biotechnology, Yuseong-gu, Daejeon, South Korea; 3 Department of Biological Science, KAIST (Korea Advanced Institute of Science and Technology), Daejeon, South Korea; French National Centre for Scientific Research - Université Aix-Marseille, France

## Abstract

In *Drosophila melanogaster*, specification of wing vein cells and sensory organ precursor (SOP) cells, which later give rise to a bristle, requires EGFR signaling. Here, we show that Pumilio (Pum), an RNA-binding translational repressor, negatively regulates EGFR signaling in wing vein and bristle development. We observed that loss of Pum function yielded extra wing veins and additional bristles. Conversely, overexpression of Pum eliminated wing veins and bristles. Heterozygotes for Pum produced no phenotype on their own, but greatly enhanced phenotypes caused by the enhancement of EGFR signaling. Conversely, over-expression of Pum suppressed the effects of ectopic EGFR signaling. Components of the EGFR signaling pathway are encoded by mRNAs that have Nanos Response Element (NRE)–like sequences in their 3’UTRs; NREs are known to bind Pum to confer regulation in other mRNAs. We show that these NRE-like sequences bind Pum and confer repression on a luciferase reporter in heterologous cells. Taken together, our evidence suggests that Pum functions as a negative regulator of EGFR signaling by directly targeting components of the pathway in *Drosophila*.

## Introduction

A variety of cellular processes such as cell fate specification, proliferation, and apoptosis utilize epidermal growth factor receptor (EGFR) signaling. Upon activation, the signaling proceeds through Drk, Sos, and Ras activation, to a phosphorylation cascade involving Raf (MAPKKK) and Dsor1 (MEK). The pathway culminates in activation of *rolled* (*rl*) MAP kinase (MAPK), which phosphorylates a suite of substrates to determine a specific cellular response [1]. Since aberrant signaling results in abnormal organ formation or tumorigenesis [2], intricate spatio-temporal regulation of the signaling is essential. Thus diverse negative regulators are employed to precisely regulate EGFR signaling.

In *Drosophila melanogaster*, adult wing blade has five wing veins, which are differentiated in the wing imaginal disc during larval and pupal stages. EGFR signaling during the larval period promotes wing vein cell differentiation. Enhanced EGFR signaling results in the development of extra-wing veins, whereas reduced signaling results in wing vein loss [3]–[10]. Apparently, the levels of EGFR signaling are carefully regulated to ensure normal vein development.

Large bristles (macrochaetes) on the notum of adult flies arise from a single sensory organ precursor (SOP) cell in the wing imaginal disc during larval development. Each SOP cell is selected from a group of equipotent cells in a proneural cluster that is specified by high level expression of proneural genes such as *achaete* (*ac*) and *scute* (*sc*) [11]–[15]. Persistent expression of proneural genes in SOP cells requires EGFR signaling [16], [17]. Reduced EGFR signaling results in loss of SOP cells in the disc and macrochaetae in the adult [4], [16]. Conversely, excess EGFR signaling evokes supernumerary SOP cells by stimulating proneural gene expression [16], in turn causing the formation of extra bristles on the thorax and notum of the adult. Thus, as in the case of wing vein specification, the selection of SOP cells from proneural clusters requires precise regulation of EGFR signaling.

In our study, we observed extra wing veins and thoracic macrochaetes in *pum* mutants, which is reminiscent of phenotypes associated with up-regulation of EGFR signaling. Our genetic interaction analysis suggests that Pum functions as a negative regulator of EGFR signaling. Pum is a translational repressor that binds to the Nanos Responsive Element (NRE) sequence at the 3’UTR of its target mRNAs [18]–[20]. We demonstrated that Pum binds to potential NRE sequence found in the EGFR, Drk, Sos, and MAPK (*rl*) 3’UTRs and represses reporters containing these NRE sequences. This study revealed a role for Pum in formation of wing veins and bristles by negatively regulating EGFR signaling.

## Results

### Genetic Interaction between Pum and EGFR Signaling in Wing Vein Formation

**Figure 1:**
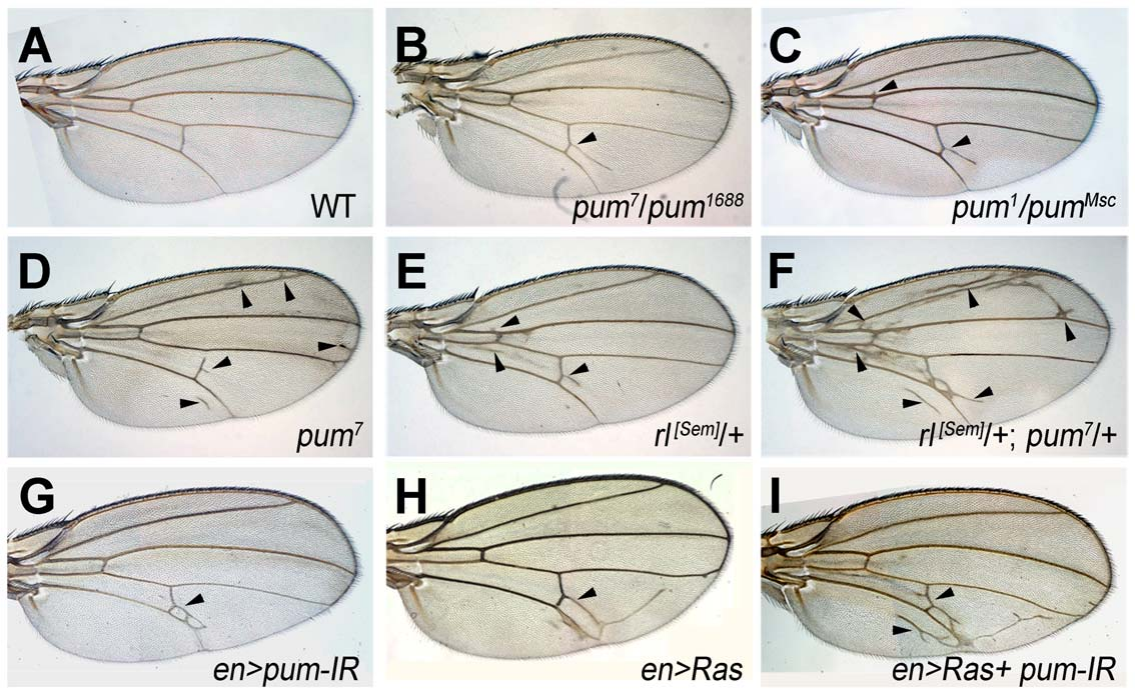
Genetic interaction between Pum and EGFR signaling in wing vein development. Wild-type wing displays five wing veins (A). Extra wing veins indicated by arrowheads arose in *pum* mutants, *pum^7^*/*pum^1688^* (B), *pum^1^*/*pum^Msc^* (C), and *pum^7^*/*pum^7^* (D). Extra wing veins arose in a gain of function mutant of *rl* (*rl^[Sem]^*/+) (arrowhead, E). *pum* dominantly increased wing veins induced by enhanced *rl* signaling (*rl^[Sem]^*/+; *pum^7^*/+) (F). Extra wing veins also arose in the posterior region where *pum* is downregulated by RNA interference (*en-Gal4*/+; *UAS*-*Pum-IR/+*) (G) or Ras is over-expressed (H). RNAi reduction of *pum* with enhanced Ras expression greatly increased wing veins (*en-gal4*/*UAS-Ras*; *UAS-Pum-IR*/+) (I).

**Figure 2:**
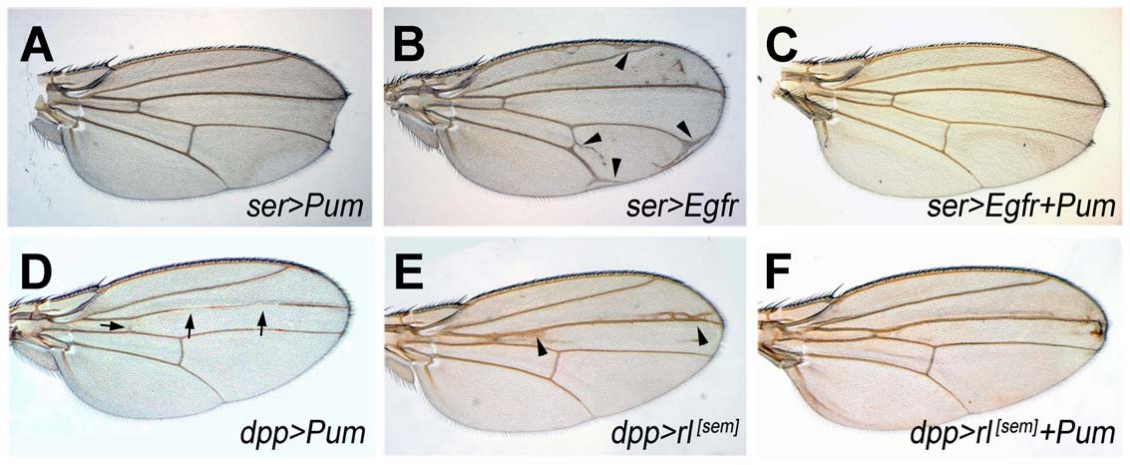
Pum overrides EGFR signaling. *ser-Gal4* derived expression of Pum and EGFR (A-C). Pum expression (*UAS-Pum*/+; *ser-GAL4*/*+*) resulted in notching in the wing boundary (A). EGFR expression (*ser-GAL4*/*UAS-EGFR)* caused extra-wing veins (arrowhead, B). Concomitant *pum* expression (*UAS-Pum*/+; *ser-GAL4*/*UAS-EGFR*) diminished extra wing veins induced by enhanced EGFR signaling and generated notch around wing boundary (C). *dpp-Gal4* derived expression of Pum and *rl^[Sem]^* (D-F). Pum expression (*UAS-Pum*/+; *dpp-GAL4*/+) eliminated wing veins (arrow, D), whereas *rl^[Sem]^* expression (*dpp-GAL4>>UAS-rl^[Sem]^*) produced extra wing veins (arrowheads in E) at the A/P boundary. Concomitant Pum expression diminished extra wing veins induced by enhanced Rl signaling (*UAS-Pum*/*UAS- rl^[Sem]^*; *dpp-GAL4*/+) (F).

We observed that, *pum^7^*/*pum^7^* and *pum^1688^*/*pum^1688^* adult “escapers” have rare extra wing veins (arrowhead in Figure 1D and data not shown). Transheterozygotes of *pum* alleles, *pum^1^*/*pum^1688^*, *pum^1^*/*pum^Msc^*, *pum^1^*/*pum^3^*, *pum^3^*/*pum^1688^*, *pum^3^*/*pum^Ms^* ^c^ and *pum^1688^*/*pum^Msc^* all displayed similar extra wing vein phenotypes (Figure 1B, 1C and data not shown). The penetrance for extra vein phenotypes is incomplete: *pum^1/Msc^* (68%, n = 54), *pum^3/Msc^* (77%, n = 175), *pum^3/1688^* (55%, n = 81), *pum^1688/Msc^* (37%, n = 45). Extra wing veins also arose where *pum* function is reduced in wing imaginal disc via RNAi of *pum* (*en-gal4*/+; *UAS-pum-IR*/+) (penetrance > 90%, n = 33); these flies displayed extra-wing veins only in the posterior compartment, where *en-GAL4* is active (arrowhead in Figure 1G). Extra wing veins also arise when EGFR/Ras/MAPK signaling is enhanced [3], [21]. For example, a gain of function mutant, *rl^[Sem]^* heterozygotes (*rl^[Sem]^*/+) with elevated MAPK activity [3], [22] produces extra vein material (Figure 1E) (penetrance >90%, n>10). In addition, wing veins arose in the posterior compartment of the wing when Ras was expressed by *en-Gal4* (*en-gal4*/*UAS-Ras*) (Figure 1H). Conversely, wing veins are lost when EGFR signaling is reduced (Figure S1) or Pum is overexpressed (Figure 2D).

We explored genetic interactions between Pum and EGFR signaling. Toward this end, we examined the effect of eliminating one copy of *pum* ^+^ on the wing vein phenotypes associated with *rl^[Sem]^*. Eliminating one copy of *pum* (*pum/+*) by itself does not produce ectopic wing vein (data not shown). However, eliminating one copy of *pum* greatly enhanced the extra wing vein phenotype in *rl^[Sem]^* flies (*rl^[Sem]^*/+; *pum^7^*/+) (arrowhead in Figure 1F) (penetrance > 90%, n >10). Likewise, greatly increased ectopic wing veins were generated when *ras* activation was combined with *pum* knock-down (*en-gal4*/*UAS-Ras*; *UAS-Pum-IR*/+) (arrow in Figure 1I). Taken together these results indicate that reduction of *pum* synergistically enhanced EGFR signaling, consistent with the idea that *pum* negatively regulates EGFR signaling in wing vein formation.

If *pum* negatively regulates EGFR signaling, over-expression of *pum* should override EGFR signaling. We tested this hypothesis and found that ectopic expression of EGFR under *ser-Gal4* control (active in the dorsal compartment during the 2^nd^ instar and the dorso-ventral boundary in the third instar) resulted in extra wing veins around the wing boundary (arrowhead in Figure 2B). Ectopic expression of Pum via *ser-GAL4* resulted in development of a distal wing notch (Figure 2A). Co-overexpresson of *pum* with EGFR (*UAS-pum*/+; *ser-GAL4*/*UAS-EGFR)* (Figure 2C) suppressed the development of ectopic veins, generating wing indistinguishable from those in which Pum alone is mis-expressed. Likewise, Co-ectopic expression of *pum* with *rl^[Sem]^* (*UAS-Pum*/*UAS-rl^[Sem]^*; *dpp^disk^-Gal4*/+) (Figure 2F) resulted in suppression of ectopic veins caused by *rl^[Sem]^* overexpression (*UAS-rl^[Sem]^*/+; *dpp^disk^-Gal4* /+) (Figure 2E). Thus, both loss- and gain- of Pum function modify EGFR pathway activity in a manner that suggests negative regulation by Pum.

### Pum Negatively Regulates EGFR/Ras/MAPK Signaling in Bristle Formation

**Figure 3:**
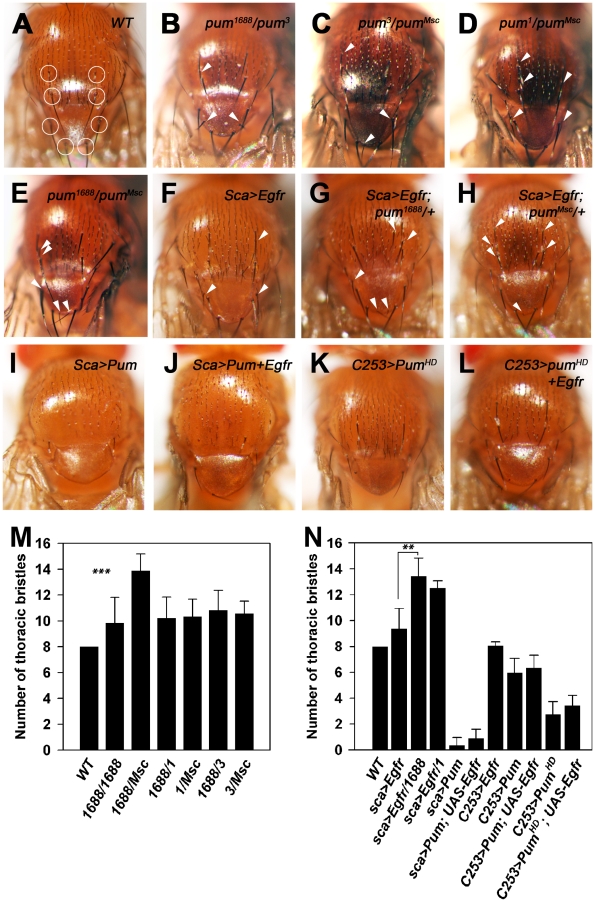
Pum negatively regulates EGFR signaling on macrochaete development. Wild-type thorax and notum bears macrochaetes at specific positions (circles, A). Extra macrochaetes indicated by arrowheads are produced in transheterozygous *pum* mutants, *pum^1688^*/*pum^3^* (B), *pum^3^*/*pum^Msc^* (C), *pum^1^*/*pum^Msc^* (D), and *pum^Msc^*/*pum^1688^* (E). Extra macrochaetes arose by ectopic expression of EGFR in SOP (*sca-GAL4*/+; *UAS-EGFR*/+) (F). One copy reduction of *pum* greatly increased extra macrochaetes induced by enhanced EGFR signaling in SOP (*sca-GAL4*/+; *UAS-EGFR*/*pum^1688^*) (G) and (*sca-GAL4*/+; *UAS-EGFR*/*pum^Msc^*) (H). Ectopic expression in SOP of full-length *pum* (*sca-GAL4*/*UAS-pum*) (I) or Puf (*C253-GAL4*/UAS-*Pum^HD^*) (K) eliminated macrochaetes. Concomitant expression in SOP of EGFR with either *pum* (*sca-GAL4*/*UAS-Pum*; *UAS-EGFR*) (J) or Puf (*C253-GAL4*/*UAS-Pum^HD^*; *UAS-EGFR*) (L) eliminated bristles. The number of macrochaetes circled in (A) was counted (M, N). ***; P value <0.001. **; P value <0.01. The P values were obtained by student’s t-test in SigmaPlot.

The wild-type notum bears macrocheates at specific position (circles, Figure 3A), whereas escapers of *pum* homozygote mutants and transheterozygotes of *pum* alleles (*pum^13^*/*pum^13^*, *pum^1688^*/*pum^1688^*, *pum^7^*/*pum^3^*, and *pum^ 7^*/*pum^1688^*) have extra macrochaetae (arrowhead in Figure 3B-E, M; Table 1). The penetrance for this phenotype ranges from 58?99%: *pum^3/Msc^* (99%, n = 175), *pum^1688/Msc^* (88%, n = 45), *pum^7/1688^* (58%, n = 24). In contrast, ectopic expression of *pum* in the SOP cells with the *sca-Gal4* driver [23] eliminated bristles (penetrance > 90%, n = 17) (Figure 3I, N; Table 1). These phenotypes are the inverse of those associated with ectopic EGFR signaling, which results in extra bristles [16]. For example, overexpression of EGFR (*UAS-EGFR*) by *sca-Gal4* induced extra bristles (Figure 3F, N; Table 1).

**Table 1:** **The number of macrochaetes reveals genetic interaction between Pum and EGFR.**

Genotype	Average number of macrochaetes *	s.d.	n	P
*w^1118^*	8.00	( ± 0.00)	(46)	
*pum^1688^*/*pum^1688^*	9.86	( ± 1.95)	(28)	<0.0001**
*pum^1^*/*pum^1688^*	10.21	( ± 1.63)	(29)	<0.0001
*pum^1^*/*pum^Msc^*	10.31	( ± 1.36)	(51)	<0.0001
*pum^1688^*/*pum^Msc^*	13.86	( ± 1.30)	(29)	<0.0001
*pum^1688^*/*pum^3^*	10.81	( ± 1.55)	(52)	<0.0001
*pum^Msc^*/*pum^3^*	10.58	( ± 0.94)	(52)	<0.0001
*sca-GAL4*/*UAS-EGFR*	9.37	( ± 1.58)	(40)	
*pum^1688^*/TM3	8.27	( ± 1.15)	(22)	
*sca-GAL4*/*UAS- EGFR*; *pum^1688^*/+	13.43	( ± 1.40)	(10)	
*pum^Msc^*/TM3	8.15	( ± 0.37)	(20)	
*sca-GAL4*/*UAS- EGFR*; *pum^Msc^*/+	12.50	( ± 0,58)	(8)	
*sca-GAL4*/*UAS-Pum*	0.35	( ± 0.61)	(17)	
*sca-GAL4*/*UAS-Pum*; *UAS- EGFR*/+	0.88	( ± 0.70)	(17)	
*C253-GAL4*/*UAS- EGFR*	8.08	( ± 0.28)	(38)	
*C253-GAL4*/*UAS-Pum*	5.97	( ± 1.11)	(60)	
*C253-GAL4*/*UAS-Pum*; *UAS-EGFR*/+	6.33	( ± 0.99)	(24)	0.167
*C253-GAL4*/*UAS-Pum^HD^*	2.73	( ± 0.98)	(26)	
*C253-GAL4*/*UAS-Pum^HD^*; *UAS-EGFR*/+	3.42	( ± 0.79)	(21)	0.013

* Bristles circled in the Figure 3A were counted. s.d., standard deviation. N, number of flies counted. P, P-value by student’s t-test.

** *pum* mutants compared to *w^1118^*.

We examined whether *pum* genetically interacts with EGFR during bristle formation. Eliminating one copy of *pum* (*pum/+*) did not affect the number of bristles by itself (data not shown), but greatly enhanced bristle number induced by ectopic expression of EGFR (*sca-Gal4*/*UAS-EGFR*; *pum/+*) (Figure 3G, H, N; Table 1). This result suggests that Pum negatively regulates EGFR signaling in bristle formation. To examine whether ectopic Pum can suppress EGFR signaling, we co-expressed Pum and EGFR (*sca-Gal4*/*UAS-EGFR*; *UAS-Pum/+*) and found that co-expression of Pum and EGFR resulted in the elimination of EGFR-induced extra bristles, phenotypes similar to Pum over-expression (Figure 3J, 3N; Table 1). These data indicate that Pum effectively down-regulate EGFR signaling in bristle formation.

The C-terminal Puf domain (or Pum homology domain, Pum^HD^) mediates Pum binding to NRE and Nos and possesses translational repressor activity [19]. Ectopic expression in SOP cells of the Puf domain *(C253-Gal4/UAS-Pum^HD^)* eliminated bristles (Figure 3K, N; Table 1). Concomitant expression of Puf with EGFR *(C253-Gal4/UAS-Pum^HD^; UAS-EGFR)* was able to suppress EGFR-induced bristle formation (Figure 3L, N; Table 1). Thus, the Puf region alone can repress EGFR signaling in the formation of bristle.

### Pum Activity in the Wing Disc

**Figure 4:**
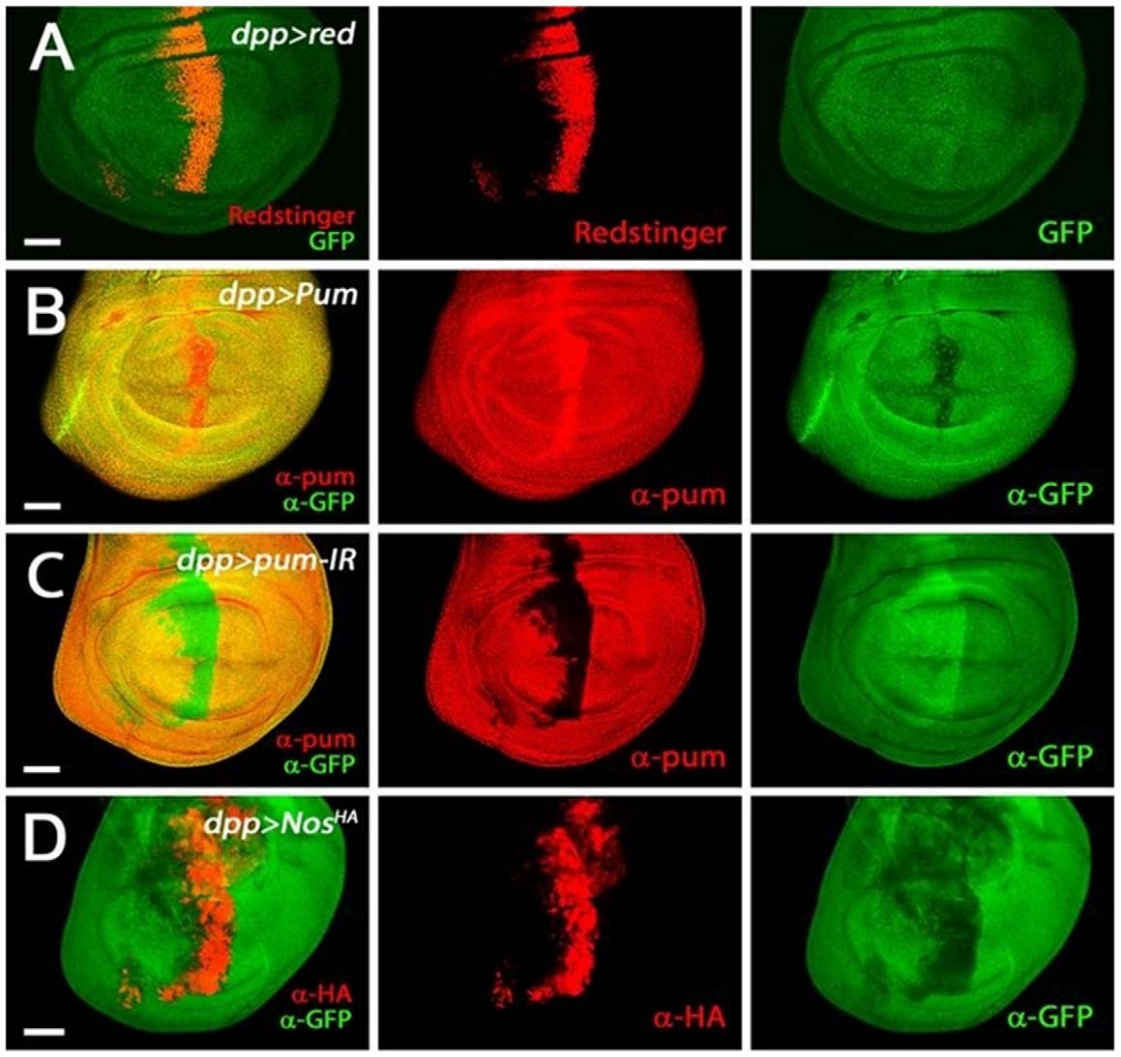
Pum activity in the wing disc. The 3^rd^ larval wing discs harboring a tub-GFP-NRE construct were visualized by GFP or redstinger fluorescence or immuno-stained by antibodies (α-GFP, α-Pum and α-HA). The *dpp-Gal4* driven expression of redstinger (A), Pum (B), Pum-IR (C), and Nos (D) were monitored, as shown in the second column. Pum activity level was monitored by GFP signals, as shown in the third column. The first column is the merged images of the second and third columns. Scale bars indicate 50 µm.

Although EGFR pathway component expression and activity have been well characterized in imaginal discs, Pum activity in the discs has not been well documented. By histochemical methods, we found that Pum is uniformly expressed in wing imaginal discs (not shown). To distinguish uniform expression from uniform background, we performed two additional experiments. Using the *dpp-GAL4* driver that is active near the anterior-posterior (A/P) compartment boundary (Figure 4A), we over-expressed either wild type Pum or Pum RNAi. As shown in Figure 4, ectopic Pum antigen is detected where *dpp-GAL4* is active in the former experiment; conversely the antigen is specifically depleted in the latter experiment. We conclude that Pum is expressed throughout the wing disc and thus available to regulate EGFR pathway components.

We also assayed Pum activity in wing discs using a GFP reporter mRNA bearing NRE sequences in its 3’-UTR [23]. Modulating the level of Pum near the A/P compartment boundary via *dpp-GAL4* regulates accumulation of GFP encoded by this reporter (Figure 4B, C) demonstrating that Pum is active in the wing disc. Furthermore, over-expression of Nos, which is a cofactor of Pum in other tissues, negatively regulates GFP (Figure 4D). Thus, Pum is both expressed and active in the wing disc.

### Pum can Repress Translation of EGFR and *rl* through Binding to NRE Sequences in their 3’-UTRs

**Figure 5:**
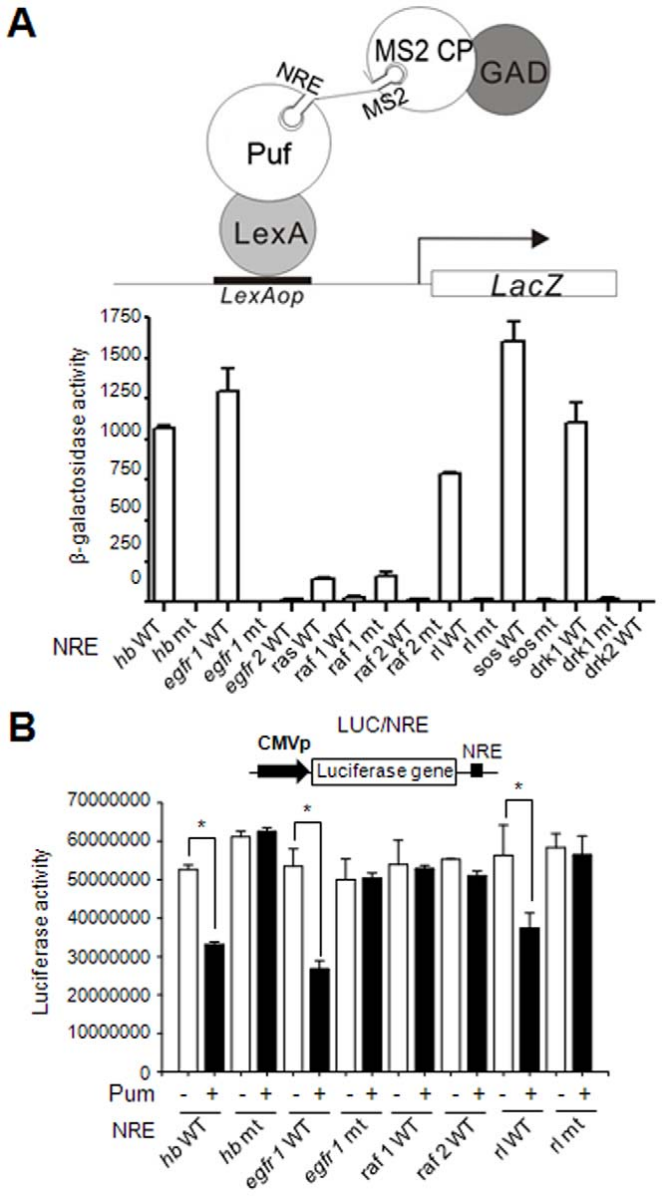
Pum binds to the potential NRE-like sequence of EGFR signaling components. (A) (Upper) Schematic drawing of the yeast three-hybrid assays. An RNA containing the NRE and MS2 sequence recruits both Gal4 transcriptional activation domain (GAD)-MS2 coat protein (CP) fusion protein (GAD-MS2 CP) and lexA DNA binding domain (DBD)-Puf fusion protein (LexA-Puf). The resultant ternary complex leads to the expression of *lacZ* coding sequence through binding to *lexA* binding sequence (LexAop). (Lower) Yeast YPH500 cells harboring the *lex*A_op_-*Lac*Z reporter, LexA DBD-Puf and GAD-MS2 CP were transformed with vectors that allow for expression of diverse NRE-MS2 transcripts as indicated. Liquid β-galactosidase assays were carried out for transformants. The mean ± SD values were obtained from at least three independent experiments and are presented on the Y-axis. (B) (Upper) Schematic diagram of a reporter containing *luciferase* (*luc*) coding sequence with CMV promoter (black arrow) and NREs (black box) in its 5′- and 3′-UTR, respectively. (Lower) HEK293 cells were transfected with the luciferase reporter plasmid containing NRE-like sequence as indicated, alone (−) or in combination with *pum* expression vector (+). Luciferase activities were measured and the mean ± SD values obtained from at least three independent experiments performed in triplicate (*, *P* < 0.05). The P-values were obtained by student’s t-test in SigmaPlot.

The genetic analysis described above indicates that Pum down-regulates EGFR signaling. Thus we searched the 3’-UTRs encoding EGFR components for potential NRE sequences (**UGUA**N(N)**AUA**, where N is any nucleotide) as a first step in determining whether regulation by Pum might be direct [24], [25] (Table 2). A genome-wide screen had previously shown that some mRNAs of EGFR pathway can be co-precipitated with Pum [24], [25]. We identified two putative NRE-like sequences in each of the EGFR, Raf, and Drk 3’-UTRs, termed NRE 1 and NRE 2; Ras, Rolled (Rl), and Sos each possess a single putative NRE-like sequence. We next examined whether Pum binds directly to these putative NREs. Using a well-characterized yeast three-hybrid assay for Pum binding [26] we found that Pum bound to the EGFR NRE1, Rl NRE, Sos NRE, and Drk NRE1 sequences (Figure 5A; Table 2). Binding was abolished by mutation of the sequence in the NRE from UGU to ACA (Figure 5A; Table 2). To investigate whether Pum binding to the NREs of EGFR, Rl, Sos, and Drk mediated translational repression, we introduced each NRE into the 3’-UTR of the *luciferase* (*luc*) gene, using wild type and mutant *hunchback (hb)* NRE sequences as controls [27]. Pum repressed translation of *luc* containing EGFR NRE1, Rl NRE, Sos NRE, and Drk NRE1; the putative Raf NRE1, which does not bind appreciably to Pum, does not mediate repression (Figure 5B; Table 2; data not shown). Mutant forms of each NRE that do not bind Pum in the yeast assay do not confer regulation by Pum in the Luciferase assay (Figure 5B; Table 2). These data indicate that Pum directly binds to various NREs in the mRNAs encoding EGFR signaling components and can repress their translation.

**Table 2:** **Pum binding and repression of NRE-like sequences of EGFR and its transducers.**

3' UTR	UGUANAUA *	Binding**	Repression***
*hb* NRE	GUCCAAAAU**UGUA**C**AUA**AGCCG	**+++**	Y
*hb* NRE mt	GUCCAAAAU**acUA**C**AUA**AGCCG	-	N
*egfr* NRE 1	CCAUAGAUU**UGUA**A**AU**UACUCU	+++	Y
*egfr* NRE 1 mt	CCAUAGAUU**acaA**A**AU**UACUCU	-	N
*egfr* NRE 2	UCCUGCGCU**UGUA**GA**AU**CCAUG	-	ND
*ras* NRE	CACGCUAUA**UGUA**U**AUA**GAUGU	-	ND
*raf* NRE 1	UUGUCCCUC**UGUA**C**AUA**AGCGA	-	N
*raf* NRE 1 mt	UUGUCCCUC**acaA**C**AUA**AGCGA	-	N
*raf* NRE 2	ACGCCCAUG**UGUA**C**AUA**ACUGC	-	ND
*raf* NRE 2 mt	ACGCCCAUG**acaA**C**AUA**ACUGC	-	ND
*rl* NRE	UAAGAAACG**UGUA**UU**AUA**UUGA	++	Y
*rl* NRE mt	UAAGAAACG**acaA**UU**AUA**UUGA	-	N
*sos* NRE	AUAAAUAUU**UGUA**A**AUA**UCGAGA	+++	Y
*sos* NRE mt	AUAAAUAUU**acaA**A**AUA**UCGAGA	-	N
*drk* NRE 1	AACUAGAUA**UGUA**A**AU**UUAUUUG	+++	Y
*drk* NRE 1 mt	AACUAGAUA**acaA**A**AU**UUAUUUG	-	N
*drk* NRE 2	GCGGCGAC**UGUA**A**AU**UGAUUAU	-	ND

* NRE consensus sequences (**UGUA**N**AUA**).

** Puf binding to NRE-like sequence determined by yeast three hybrid assay as shown in Figure 5A. +++, strong binding; ++, moderate binding.

*** Pum repression of a reporter containing NRE-like sequence as determined in Figure 5B. Y, repression; N, no repression; ND, not determined.

## Discussion

We have shown that, in the absence of Pum, extra bristles and wing veins develop, while over-expression of Pum eliminates bristles and wing veins. Several lines of evidence show that the role of Pum is to negatively regulate development of wing veins and bristles. First, loss- and gain-of Pum function produced aberrant wing vein and bristle phenotypes that are inverse to those produced by altered EGFR signaling. Second, reduction of Pum activity greatly enhanced phenotypes associated with reduced EGFR signaling. Third, concomitant expression of Pum suppressed phenotypes associated with ectopic EGFR signaling. In support of the genetic conclusion, we show that Pum binds the NRE-like sequence of EGFR, Rl, Sos, and Drk mRNAs and represses translation of a reporter containing these sequences in heterologous cells, suggesting that Pum is a negative regulator of EGFR signaling.

To define Pum’s role in the development of wing veins and bristles precisely, we attempted to locate Pum protein and measure Pum activity through a GFP-NRE construct in the 3^rd^-instar larval and pupal wing imaginal discs where wing vein and SOP cells are specified. We obtained a low- level ubiquitous expression of Pum and broad Pum activity, suggesting that Pum might function as general attenuator of EGFR signaling.

Our discovery of negative regulation of EGFR signaling by Pum is not confined to *Drosophila* somatic cells, since it has also been reported in germline cells of *C. elegans*, cultured human stem cells, and yeast cells [28], [29]. Thus, it is likely that Pum regulation of EGFR signaling is universal and involves diverse developmental contexts, ranging from *C. elegans* to *Drosophila* and humans.

## Materials and Methods

### *Drosophila* Strains

The *pum* alleles were used as follow; *pum^1688^*, *pum^Msc^*, *pum^1^*, *pum^3^* and *pum^7^* [30]–[34]. The *UAS-Pum^HD^* flies (a gift from Y. Jan) can drive expression of the RNA binding region of Pum (1092–1427) [35]. *UAS-Pum-IR*, *UAS-EGFR-IR*, *UAS-Ras-IR* and *UAS-Raf-IR* (in vivo RNA interference) lines were obtained from VDRC RNAi library [36]. *UAS-Nos^HA^* line was obtained from H. Lin. EGFR pathway alleles and transgenes were used as follows: *rl^[Sem]^* [3]; *UAS-EGFR*, *UAS-rl^[Sem]^* [37], *UAS-Ras* [9]; Gal4 lines, *sca-Gal4* and C*253-Gal4* drive expression in proneural clusters [15], [16], [38]; *dpp^disk^-Gal4* [39], *ser-Gal4*, and *en-Gal4* drive expression in a stripe at the anterior-posterior boundary, dorso-ventral boundary, and in the posterior compartment of wing pouch, respectively. They are described in detail at FlyBase (http://flybase.bio.indiana.edu). *UAS-Pum* lines were constructed as follows; the full-length Pum coding sequence was removed from pOT2-*pum* (LD44635) (Drosophila Genomics Resource Center, Bloomington, IN) via digestion with *Eco*RI, *Xba*I digestion. The ends were filled using Klenow fragment. The *pum* sequence was then cloned into the *Xho*I site (blunted with Klenow) of pINDY5 [40] to create *UAS-Pum*. The construct was verified by DNA sequencing. Five independent transgenic flies were produced and used in this study.

### Yeast Three-hybrid Assays

The C-terminal Puf region (1093∼ 1427) of the *Drosophila* Pum was generated from the LD44635 by polymerase chain reaction (PCR) and cloned into *Eco*RI/*Xho*I sites of pACT2 AD vector (Clontech) to produce Gal4 transcriptional activation domain (GAD)-Puf (GAD-Puf). The NRE and its mutant fragments were generated by oligomer dimerization, and inserted into the *Sma*I/*Spe*I sites of pIIIA/MS2-2 to express NRE-MS2 RNA for three-hybrid test [41]. The sequences of the primers were shown in the Table 2. All constructs were verified by DNA sequencing. pIII/MS2/*hb* NRE and pIII/MS2/*hb* NRE mt produce *hb* NRE-MS2 RNA and *hb* NREmt-MS2 RNA, respectively. Yeast strain, YPH-500 (MATα, *ura3-52, lys 2-80, ade2-101, trp1-*Δ*63, his3-*Δ*200, leu2-*Δ*1*), was used to analyze RNA-protein interaction and three-hybrid assays were performed as described [41]. In brief, plasmid expressing the LexA-Puf fusion protein, GAD-MS2 coat protein, and an NRE-MS2 RNA were co-transformed into yeast strain YPH-500 harboring the lexAop-LacZ reporter. Liquid assays for *β-galactosidase* activity of three or more transformants were carried out as described [27], [42].

### Luciferase Reporter Assays

A full-length Pum from LD44635 was cloned into the *Eco*RI/*Xho*I sites of pcDNA3 (Invitrogen) by PCR. The same NRE fragments used for the yeast three-hybrid assay were inserted into the *Bam*HI/*Xho*I sites of pcDNA3-LUC [27]. HEK293 cells were maintained with Dulbecco’s modified Eagle’s medium (DMEM) supplemented with 10% fetal bovine serum for 24 h and transiently transfected with the appropriate set of reporter and expression plasmids using SuperFect reagent (Qiagen). For reporter assays, 24 h after transfection, cells were harvested and assayed for luciferase activity as described previously [27], [42]. The results from triplicate samples were averaged and normalized to *LacZ* expression from pSV-β-gal (Promega). The plasmid DNAs used for transfection included the reporter plasmid having NRE wild-type or its mutant, the pSV-β-gal control plasmid, and pcDNA3-*pum*.

### Immunostaining of the 3rd Instar Larval Wing Disc

The 3rd instar larval wing discs were dissected in PBS. The tissues were fixed for 15 minutes with gentle rocking in 4% formaldehyde in PBS. After fixation, the tissues were washed three times in PBT (PBS, 0.1% Tween-20) at RT for 15 minutes. The tissues were then blocked for 1 hour by 5% normal Goat serum in PBT. Primary antibodies were incubated overnight at 4°C. The wing discs were then washed four times with PBT for 10 minutes, and incubated for two hours with secondary antibodies, then washed and mounted in a VectaShield Mounting medium(Vector Laboratories). The following antibodies were used (dilutions noted in parentheses): Rabbit anti-GFP (1:1000) (Molecular Probes); rat anti-Pum (1:200, a gift from Dr. MacDonald); and monoclonal anti-HA (12CA5, 1:500) (Roche). Fluorescence-conjugated secondary antibodies were used as follows: Anti-rabbit Alexa 488 conjugated (1:200); anti-rat Rhodamine conjugated (1:200); and anti-mouse Alexa 568 conjugated (1:200) (molecular Probes). The stained images were processed via the LSM 510 confocal microscope (Zeiss).

## Supporting Information

Figure S1 **Reduction of EGFR signaling causes loss of wing veins.** Wing veins are lost by the reduction of EGFR signaling (*en-GAL4/UAS-EGFR-IR* (A), *en-GAL4/+; UAS-Ras-IR/*+ (C), *en-GAL4/UAS-Raf-IR* (E)). Concomitant reduction of Pum does not overrule vein loss by reduced EGFR signaling (*en-GAL4/UAS-EGFR-IR*; *UAS-Pum-IR*/+ (B), *en-GAL4/+; UAS-Pum-IR/UAS-Ras-IR* (D), *en-GAL4/UAS-Raf-IR*; pum-IR/+ (F)).
(TIF)
