# Supplementary figures and images for: Negative Regulation of EGFR/MAPK Pathway by Pumilio in Drosophila melanogaster
Source: PLoS One. 2012 Apr 13;7(4):e34016. doi: 10.1371/journal.pone.0034016 (PMC3326002; doi:10.1371/journal.pone.0034016)

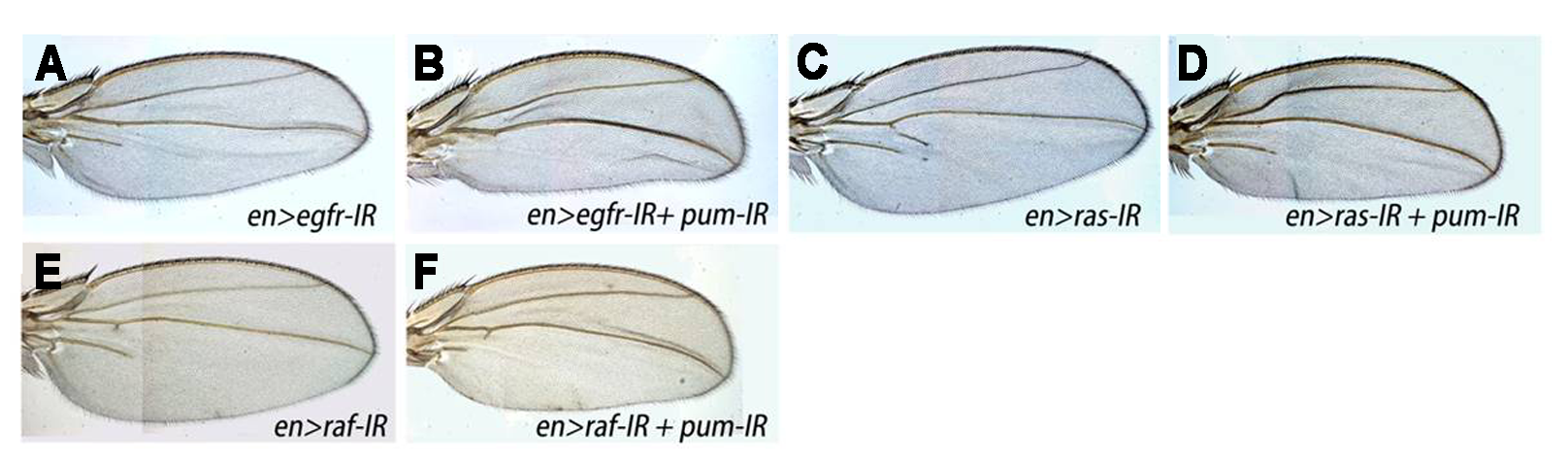

Supplement: Figure S1 — Reduction of EGFR signaling causes loss of wing veins. Wing veins are lost by the reduction of EGFR signaling (en-GAL4/UAS-EGFR-IR (A), en-GAL4/+; UAS-Ras-IR/+ (C), en-GAL4/UAS-Raf-IR (E)). Concomitant reduction of Pum does not overrule vein loss by reduced EGFR signaling (en-GAL4/UAS-EGFR-IR; UAS-Pum-IR/+ (B), en-GAL4/+; UAS-Pum-IR/UAS-Ras-IR (D), en-GAL4/UAS-Raf-IR; pum-IR/+ (F)). (TIF) [file pone.0034016.s001.tif]
